# Null Genotypes of *GSTM1* and *GSTT1* Contribute to Risk of Cervical Neoplasia: An Evidence-Based Meta-Analysis

**DOI:** 10.1371/journal.pone.0020157

**Published:** 2011-05-23

**Authors:** Lin-Bo Gao, Xin-Min Pan, Li-Juan Li, Wei-Bo Liang, Peng Bai, Li Rao, Xiao-Wei Su, Tao Wang, Bin Zhou, Yong-Gang Wei, Lin Zhang

**Affiliations:** 1 Laboratory of Molecular and Translational Medicine, West China Institute of Women and Children's Health, West China Second University Hospital, Sichuan University, Chengdu, Sichuan, People's Republic of China; 2 Department of Forensic Pathology, Henan University of Science and Technology, Luoyang, Henan, People's Republic of China; 3 Department of Forensic Biology, West China School of Preclinical and Forensic Medicine, Sichuan University, Chengdu, Sichuan, People's Republic of China; 4 Department of Cardiology, West China Hospital of Sichuan University, Chengdu, Sichuan, People's Republic of China; 5 Department of General Surgery, West China Hospital of Sichuan University, Chengdu, Sichuan, People's Republic of China; Marienhospital Herne - University of Bochum, Germany

## Abstract

**Background and Objectives:**

Glutathione S-transferases (GSTs) are multifunctional enzymes that play a key role in the detoxification of varieties of both endogenous products of oxidative stress and exogenous carcinogens.

**Methods:**

In this meta-analysis, twenty-five studies were identified by searching PubMed, EMBASE, ISI Web of Science and CBM databases: 23 evaluated *GSTM1* and 19 evaluated *GSTT1*. Crude odds ratios with corresponding 95% confidence intervals were used to estimate the association between *GSTM1* and *GSTT1* polymorphisms and risk of cervical neoplasia. Subgroup analyses were conducted by pathological history, ethnicity, source of DNA for genotyping, quality score, and matching variable.

**Results:**

The null genotypes of *GSTM1* and *GSTT1* polymorphisms were associated with a significantly increased risk of cervical neoplasia (for *GSTM1*: OR = 1.40; 95%CI, 1.19–1.65; for *GSTT1*: OR = 1.30; 95%CI, 1.05–1.62, respectively). Subgroup analyses showed that the null genotype of *GSTM1* increased the risk of cervical neoplasia in Asians, studies with DNA isolation from white blood cells and tissue samples, both high and low quality studies, and matched studies. In *GSTM1*-*GSTT1* interaction analysis, individuals with dual null genotype were associated with a significantly increased risk of cervical neoplasia (OR = 1.72; 95%CI, 1.18–2.51).

**Conclusion:**

These findings indicate that *GSTM1* and *GSTT1* polymorphisms, particularly *GSTM1*-*GSTT1* interaction, may play critical roles in the development of cervical neoplasia. A conservative manner should be adopted to interpret these results because of obvious heterogeneity between-study, unadjusted data, and relatively small sample size in this meta-analysis. Well designed studies with larger sample size are of great value to confirm these results.

## Introduction

Cervical cancer is the second most frequent cancer among women worldwide, with approximately 493,000 new cases diagnosed and 274,000 deaths occurring each year (2002 estimates) [1]. Despite substantial declines in the incidence and mortality of cervical cancer in developed countries, more than 80% of all cervical cancer occurs in developing countries [1], [2]. The burden of cervical cancer is not only the high incidence rates in women in some developing countries, but also the societal impact because a fraction of patients who suffered from the disease in their 30's or 40's are still raising or supporting families.

It is well established that human papilloma virus (HPV) infection is a necessary but insufficient event for the development of cervical cancer [3]–[6], because not all HPV-infected patients do develop cervical cancer. Therefore, many research efforts were taken to identify cofactors for cervical cancer development. To date, the major risk cofactors have been confirmed by large meta-analysis, including smoking, multiple sexual partners, increasing parity, earlier age at first intercourse (≤20 years), and long duration of oral contraceptive use [7]–[9]. However, it is currently accepted that the development of cervical cancer is the result of complex interaction of both environmental and genetic factors [10]. Epidemiological evidence has shown that there is a significant familial clustering among biological relatives. The familial relative risk for individuals with biological full-sisters of cervical cancer cases is almost twice as high as those with biological full-sisters of controls [11]–[13]. Recently, several meta-analysis studies revealed that a polymorphic variant of the tumor suppressor *P53* (Pro72Arg) may represent a genetic marker for cervical carcinogenesis [14]–[17].

Over the past decades, glutathione S-transferases (GSTs) genetic variants have been explored extensively as a predictive factor for cancer prognosis [18]. GSTs are a family of enzymes with a crucial function in the detoxification of a variety of both endogenous products of oxidative stress and exogenous carcinogens [19], [20]. In humans, GST super-family consists of many cytosolic, mitochondrial, and microsomal proteins. The cytosolic family has been assigned to eight distinct classes: alpha, kappa, mu, omega, pi, sigma, theta, and zeta [21]. The mu class of GSTs, encoded by the *GSTM1* gene, is located on the short arm of chromosome 1 (1p13.3) [22]. The theta class of GSTs, encoded by the *GSTT1* gene, is locate on the long arm of chromosome 22 (22q11.23) [23]. Both *GSTM1* and *GSTT1* gene exhibit an inherited homozygous deletion polymorphism (null genotype) that is associated with an absence of enzyme activity. Individuals with homozygous deletion polymorphism are considered to be at increased risk for malignancies due to reduced efficiency in protection against environmental carcinogens [18], [24].

In 1994, Warwick et al. explored for the first time the association between *GSTM1* and *GSTT1* polymorphisms and the risk of cervical neoplasia, and found that the combination of three factors (i.e., *GSTM1* or *GSTT1* null, CYP2D6 EM, and smoking) appeared significantly different frequency in cases and controls [25], [26]. Subsequently, a large number of epidemiological studies have been addressed to evaluate the association between *GSTM1* and *GSTT1* homozygous deletion polymorphisms and risk of cervical neoplasia in diverse ethnicities [25]–[49]. However, this issue remains controversial because of inconsistent results among different studies. The possibilities for this discrepancy may be that some positive results might occur by chance and some negative findings might be caused by insufficient statistical power with small sample size. Additionally, different experimental design and selection bias should also be considered.

In order to provide strong evidence of the effects of *GSTM1* and *GSTT1* polymorphisms on cervical neoplasia risk, we carried out a quantitative meta-analysis by combining data from all published case-control studies. Additionally, gene-gene and gene-environment interactions have also been examined in this meta-analysis.

## Materials and Methods

### Selection of Published Studies

We identified all publications by conducting computer-based searches of PubMed, EMBASE, ISI Web of Science, and CBM databases without language restrictions, using the following search algorithm: (“cervical cancer” or “cervical carcinoma” or “uterine cervix cancer” or “CC” or “cervical neoplasia”) and (“glutathione S-transferase” or “GST” or “GSTM” or “GSTM1” or “GSTT” or “GSTT1”) and (“polymorphism” or “polymorphisms” or “variant”). The literature search was performed up to Aug 2010. The inclusion criteria were: (a) case-control studies that investigated the association between *GSTM1* and/or *GSTT1* polymorphism and risk of cervical neoplasia; (b) presenting original data for the calculation of odds ratios (ORs) with corresponding 95% confidence intervals (95%CIs).

One hundred and fifty-four articles were identified by searching PubMed, EMBASE, ISI Web of Science and CBM databases. Eighty-four studies were excluded after screening the title or abstract (67 were not cervical cancer; eight were no polymorphism; six were not human studies; three were not case-control studies). Then full-text articles were retrieved for assessment in detail. Forty-three were excluded with reasons for not cervical cancer (n = 23), no *GSTM1* and/or *GSTT1* polymorphism (n = 13), no available data (n = 5) and review articles (n = 2). Additionally, we excluded two studies because the results were duplicated in subsequent publications [50], [51]. Finally, a total of 25 studies were included in this meta-analysis (Figure S1). Of the 25 studies, 23 studies investigated the association between *GSTM1* polymorphism and risk of cervical neoplasia and 19 studies investigated the association between *GSTT1* polymorphism and risk of cervical neoplasia.

The groups of pathologic type were set according to the report by Klug et al. [17]. Briefly, the selected studies in this analysis were composed of unclear type of cervical cancer, squamous cell carcinoma (SCC), adenocarcinoma (AC), adenosquamous carcinoma, high-grade lesions (HGL, containing high-grade squamous intraepithelial lesions and cervical intraepithelial lesions grades 2 and 3) and low-grade lesions (LGL, containing low-grade squamous intraepithelial lesions and cervical intraepithelial lesions grade 1).

This study was approved by the ethics committee of Sichuan University. The data included in this study was taken from literatures, and thus written consent given by the patients was not needed.

### Data Extraction

Two independent researchers (Gao and Pan) extracted raw data according to the inclusion criteria. The following information was collected from each study using a data extraction form: the surname of the first author, date of publication, country of origin, year of sample collection, ethnicity, characteristics of cases and controls, DNA source for genotyping, matching variables, number of cases and controls, genotype distribution of cases and controls, and quality control for genotyping assay. Additionally, we extracted, if available, the genotype frequency of cases and controls based on age (>40 or ≤40 years), smoking status (smoking or non-smoking) and HPV infection status (HPV positive or HPV negative). Given that there was no distribution of null/present heterozygote in each single study selected, the Hardy-Weinberg equilibrium (HWE) test could not be conducted. To ensure the accuracy of data extraction, the original extraction information was checked by Li, and discordant results were settled through discussion among the three authors.

### Quality Score Evaluation

Three investigators (Gao, Pan, and Li) independently assessed the quality of included studies based on a predetermined rating scale (Table S1) that was amended from previous studies [52]–[54]. Any discrepancies were resolved by consultation with the other authors in the team group, and an ultimate decision was made by the majority of the votes cast. A numerical score ranging from 0 to 12 was assigned as a quantitative measure of literature quality. Studies were categorized as “high quality” if the quality score was ≥7; otherwise, studies were categorized as “low quality”.

### Statistical Analysis

We used crude ORs with corresponding 95% CIs as a measure of the association between *GSTM1* and *GSTT1* polymorphisms and risk of cervical neoplasia. Study-specific ORs comparing null genotype versus present genotype were combined using random-effects model (the DerSimonian and Laird) or fixed-effects model (the Mantel–Haenszel method), which was determined by the *Q*-test and *I* ^2^ statistics [55], [56]. If the *P* value for heterogeneity was ≤0.10 or *I* ^2^≥50%, indicating a high extent of heterogeneity between studies, we used the DerSimonian and Laird method to evaluate the summary ORs. In contrast, if the *P* value for heterogeneity was >0.10 and *I* ^2^<50%, indicating an absence of heterogeneity between studies [57], [58], we used the Mantel–Haenszel method to evaluate the summary ORs.

Subgroup analyses were conducted by pathological history (squamous cell cervical carcinoma, adenocarcinoma and adenosquamous carcinoma, cervical cancer of unknown type, HGL, LGL, and mixed), ethnicity (Asian, Caucasian, and mixed), source of DNA for genotyping (white blood cells, exfoliated cervical cells, tissue sample, and mixed), quality score (high versus low), matching according to age (matched versus unmatched), smoking status (smoking versus non-smoking) and HPV infection status (HPV positive versus HPV negative). Additionally, we evaluated the effect of the *GSTM1-GSTT1* interaction on cervical neoplasia compared with null/null versus present/present, null/null versus present/null, null/null versus null/present, null/present versus present/null, null/present versus present/present, and present/null versus present/present.

Logistic meta-regression was used to investigate possible sources of heterogeneity across studies. To determine the reliability of the outcomes in the meta-analysis, a sensitivity analysis was performed by exclusion an individual study each time. An evaluation of publication bias was carried out with funnel plot for visual inspection and Egger's regression asymmetry test [59]. All analyses were conducted in STATA software, version 10.0 (STATA Corp., College Station, TX).

## Results

### Characteristics of Studies

The baseline characteristics of the included studies are shown in Tables S2 and S3.

#### *GSTM1* Polymorphism

Totally, 23 studies met the inclusion criteria and were selected in this meta-analysis with 2,610 cases and 3,084 controls. Cases consisted of 32.5% patients with cervical cancer (histology not specified), 31.9% patients with SCC, 14.2% patients with HGL, 8.6% patients with LGL, 5.0% patients with SIL (unknown grade), 4.7% patients with both ICC and HGL and 3.0% patients with AC. Most of the controls (86.8%) were normal participants. There were sixteen studies of Asians, six studies of Caucasians, and two studies of mixed ethnicities that included more than one ethnicity. DNA used for *GSTM1* genotyping was extracted from white blood cells in 15 studies (62.5%). 17 studies (70.8%) mentioned genotyping quality control methods, mainly using an internal control. However, only six studies (25.0%) reported smoking status and only four studies (16.7%) detected HPV infection status.

#### *GSTT1* Polymorphism

A total of 19 studies were included in the meta-analysis with 2,092 cases and 2,054 controls. Cases consisted of 27.1% patients with cervical cancer (histology not specified), 30.5% patients with SCC, 15.9% patients with HGL, 8.6% patients with LGL, 6.3% patients with SIL (unknown grade), 5.9% patients with both ICC and HGL and 5.8% patients with AC. Most of the controls (85.5%) were normal participants. Twelve studies were conducted in Asia; four in Europe; two in America and one in South America. Similar to *GSTM1* polymorphism, most studies (68.4%) mentioned genotyping quality control methods, but only about 20% studies reported smoking status and HPV infection status.

### Meta-analysis of *GSTM1* Polymorphism and Cervical Neoplasia

The evaluations of the association between *GSTM1* polymorphism and cervical neoplasia risk are summarized in Table S4.

The null genotype of *GSTM1* polymorphism was associated with a significantly increased risk of cervical neoplasia when compared with present genotype (OR = 1.40; 95%CI, 1.19–1.65). When stratified by pathologic types, significantly elevated risks were observed in unknown type of cervical cancer (OR = 1.54; 95%CI, 1.16–2.04) and mixed group (OR = 1.98; 95%CI, 1.46–2.68) but not in groups of SCC, HGL, LGL and AC. In the subgroup analysis by ethnicity, significantly increased risks were observed in Asian population (OR = 1.60; 95%CI, 1.29–1.98) but not in Caucasian and mixed populations (Figure S2). Subgroup analysis on the basis of DNA source showed that the increased risks were found in studies that DNA was extracted from white blood cells (OR = 1.29; 95%CI, 1.08–1.55) or tissue sample (OR = 3.14; 95%CI, 1.90–5.19). No excess risk was found in studies that DNA was extracted from exfoliated cervical cells. Subgroup analysis was also performed according to quality criteria. The combined results showed that the null genotype was associated with an increased risk of cervical neoplasia in studies whether the quality score was high (OR = 1.31; 95%CI, 1.06–1.62) or low (OR = 1.49; 95%CI, 1.16–1.91). The increased risks were also found in studies in which controls were frequency matched to cases by age (OR = 1.54; 95%CI, 1.22–1.95), but not in studies in which controls were unmatched to cases by age. Additionally, subgroup analysis by age presented the results that the null genotype was associated with an increased risk of cervical neoplasia in studies with patients of age ≤40 years (OR = 2.02; 95%CI, 1.30–3.14).

### Meta-analysis of *GSTT1* Polymorphism and Cervical Neoplasia

The evaluations of the association of *GSTT1* polymorphism and cervical neoplasia risk are listed in Table S5.

The null genotype of *GSTT1* polymorphism was associated with a significantly increased risk of cervical neoplasia (OR = 1.30; 95%CI, 1.05–1.62) and unknown type of cervical cancer (OR = 1.49; 95%CI, 1.02–2.19), while the association was not observed in subgroup analyses according to ethnicity, DNA source, and quality criteria (Figure S3).

### Meta-analysis of *GSTM1-GSTT1* Interaction with Cervical Neoplasia

The evaluations of the association between *GSTM1-GSTT1* interaction and cervical neoplasia risk are shown in Table S6.

The dual null genotype was associated with a significantly increased risk of cervical neoplasia when compared with the dual present genotype (OR = 1.72; 95%CI, 1.18–2.51) (Figure S4). No significantly increased risk was detected in any other comparison group.

### Interaction between *GSTM1* and *GSTT1* and Environmental Exposure

There were six literatures which investigated the impact of interaction between *GSTM1* polymorphism and smoking on cervical neoplasia, and there were four literatures which investigated the impact of interaction between *GSTT1* polymorphism and smoking on cervical neoplasia. The effect of interaction between *GSTM1* polymorphism and HPV infection status on cervical neoplasia was reported in four studies, and the effect of interaction between *GSTT1* polymorphism and HPV infection status on cervical neoplasia was reported in five studies. No increased risks were found in the interaction between *GSTM1* and *GSTT1* polymorphisms and environmental exposure (i.e., smoking status and HPV infection status) (Tables S4 and S5).

### Heterogeneity Analysis

The findings of Q-tests and *I* ^2^ statistics were shown in Tables S4, S5, and S6. Significant heterogeneity across studies was present in overall analyses (for *GSTM1*, *I* ^2^ = 53.3%; for *GSTT1*, *I* ^2^ = 59.1%) and subgroup analyses. We explored several possible sources of the between-study heterogeneity, including cancer type, ethnicity, sample size, DNA source for genotyping and quality score. However, none of these variables could explain the heterogeneity.

### Sensitivity Analysis and Publication Bias

To assess the effect of individual study on the overall meta-analysis estimate, we excluded one study at a time, and the exclusion of any single report did not alter the significance of the final decision, suggesting that the outcomes were robust. Funnel plot and Egger's test were used to assess publication bias of literatures on cervical neoplasia. No evidence of publication bias was observed in all comparison groups (*P*>0.05).

## Discussion

The glutathiones S-transferases (GSTs) are the most important parts of phase II superfamily of metabolism enzymes. In humans, there are several GST classes that were encoded by distinct gene families [21]. Among them, *GSTM1* and *GSTT1* should be pointed out because a polymorphic deletion of these genes may influence the enzyme activity, and eventually increased vulnerability to genotoxic damage [60], [61]. Based on these backgrounds, the association has been intensively investigated between *GSTM1* and *GSTT1* polymorphisms and risk of cervical neoplasia [25]–[49]. Unfortunately, most of the studies have only a few hundred of participants, even less, which is too small to evaluate the overall effects precisely. Meta-analysis has been considered to be a powerful tool to overcome this problem by combining the results from independent studies together. In this meta-analysis, we found that the null genotypes of *GSTM1* and *GSTT1* polymorphisms were associated with a significantly increased risk of cervical neoplasia, suggesting that *GSTM1* and *GSTT1* polymorphisms may be involved in the development of cervical neoplasia. Notably, the between-study heterogeneity was observed in both overall analyses and some subgroup analyses, further studies therefore are warranted to confirm these findings.

After subgroup analysis according to ethnicity, significantly increased risks were observed in Asian population but not in Caucasian and mixed populations. The possibilities of the conflicting results among diverse ethnicities may be that the *GSTM1* and *GSTT1* polymorphisms have different effects on the risk of cervical neoplasia in different genetic backgrounds and environment which they exposed to. The major difference in the distribution of *GSTM1* and *GSTT1* polymorphisms has been reported among control groups in 2001. The frequency of *GSTM1* null genotype was 53.1% (42.0–60.0%) in Caucasians, 52.9% (42.0–54.0%) in Asians, and 26.7% (16.0–36.0%) in Africans. The frequency of *GSTT1* null genotype was 19.7% (13.0–26.0%) in Caucasians and 47.0% (35.0–52.0%) in Asians [62]. Additionally, the small sample size should also be taken into consideration because limited sample size may have not enough statistical power to detect a real effect or generate a fluctuated estimation. At present, limited studies investigated the association between *GSTM1* and *GSTT1* polymorphisms and the risk of cervical neoplasia in Caucasian and mixed populations. Therefore, well-designed studies with thousands of sample size are of great value to confirm this finding in Caucasians and other ethnic populations.

When stratified based on the source of DNA for *GSTM1* and *GSTT1* genotyping, the null genotype of *GSTM1* significantly increased the cervical neoplasia risk in studies that the polymorphism was determined from white blood cells rather than from exfoliated cervical cells. The difference of studies with DNA isolation from exfoliated cervical cells tended to be significant (*P* = 0.057). It is likely that the potentially negative results were caused by small sample size with only 299 cases and 405 controls available in this meta-analysis. Another important issue is the source of cell used for DNA analysis. DNA isolation from different cell types may influence performance of genotyping, and eventually, lead to the conflicting results.

It is absolutely pivotal for a meta-analysis to assess the quality of literatures included. Currently, no standard quality score method was developed to evaluate observational case-control studies. We used a self-made rating scale for quality assessment, which was modified from previous studies [52]–[54]. Studies included in this meta-analysis were classified into high quality (≥7) or low quality (<7) according to the quality score. The combined results showed that the null genotype of *GSTM1* polymorphism was associated with an increased risk of cervical neoplasia in both high quality studies and low quality studies. However, there was lack of association between *GSTT1* polymorphism and cervical neoplasia risk either in high quality studies or in low quality studies. These findings denote that *GSTM1* plays much more important roles than *GSTT1* in the development of cervical neoplasia.

The hypothesis of cigarette smoking being a risk factor for cervical cancer was originally presented in 1977 [63]. Subsequently, amounts of epidemiological studies reported the support for this hypothesis [7], [64]–[69]. Despite the mechanism that tobacco smoking increase the risk of uterine cervical cancer remains unknown, it is believed that the occurrence of tobacco-initiated DNA damage in the cervical epithelium may be responsible for malignant transformation [70]. Tobacco smoke contains over fifty known carcinogens, such as polynuclear aromatic hydrocarbons, aromatic amines, nicotine, and nitrosamine 4-(methylnitrosamino)-1-(3-pyridyl) -1-butanone (NNK) [70]–[73]. The concentrations of NNK in cervical mucous of cigarette smoking women were three times higher than those in non-smokers [70]. Such carcinogens may promote cancer through the stimulation of cell division or impairment of local immunosurveillance in the cervical epithelial tissue [67], [74]. In view of the crucial role that the smoking play in the etiology of cervical cancer, the effect of the interaction of *GSTM1* and *GSTT1* polymorphisms and smoking on the development of cervical neoplasia has been conducted in several studies [31]–[33], [37], [38], [46]. Therefore, it is necessary to analyze quantitatively the association between gene-environment interaction and the risk of cervical neoplasia using a meta-analysis. However, no evidence of correlation was observed between *GSTM1* and *GSTT1* polymorphisms and cervical neoplasia in combination with smoking habit. There may be a high risk of false negative results due to insufficient statistical power with very limited subjects eligible in this meta-analysis (for *GSTM1* polymorphism: 737 cases and 704 controls; for *GSTT1* polymorphism: 403 cases and 373 controls).

Persistent HPV infections are known to be the major cause of cervical cancer [5], [6]. Therefore, HPV infection status was also examined in subgroup analysis. Nevertheless, we failed to find any association between *GSTM1* and *GSTT1* polymorphisms and cervical neoplasia risk in either HPV positive women or HPV negative women. The null result may be owing to limited relevant studies included in this meta-analysis. Thus, large-scale prospective cohort studies are needed to provide the best evidence for the impact of interaction of gene-environment on the risk of cervical neoplasia.

Over the past decades, a large number of meta-analyses have been done to investigate the association between *GSTM1* and *GSTT1* polymorphisms and various cancers, including brain tumors [75], hepatocellular carcinoma [76], [77], colorectal cancer [78], [79], gastric cancer [80]–[84], breast cancer [85]–[90], bladder cancer [91]–[93], lung cancer [94]–[99], esophageal cancer [100], [101], prostate cancer [102], [103], nasopharyngeal carcinoma [104], head and neck cancer [105], oral and laryngeal cancer [106]–[108], and acute leukaemia [109], [110]. During revision of the manuscript, a similar report investigating the association between *GSTM1* and *GSTT1* polymorphisms and cervical cancer risk was published [111]. In the report, Economopoulos et al. identified publications by a search of Medline database (last search: August 3, 2009) and found that the *GSTM1* polymorphism but not *GSTT1* polymorphism was associated with the risk of cervical cancer [111]. In this meta-analysis, the eligible studies were identified by computer-based searches of three additional databases (i.e., EMBASE, ISI, and CBM) besides Medline, and the last search was performed up to August 2010. Moreover, studies examining the association between *GSTM1* and *GSTT1* polymorphisms and cervical intraepithelial neoplasia were also selected. Much more eligible studies, therefore, were included in this meta-analysis. Consistent with the results reported by Economopoulos et al., we found that the null genotype of *GSTM1* polymorphism was associated with a significantly increased risk of cervical neoplasia. Inconsistent with the results reported by Economopoulos et al., we found an evidence of an association between *GSTT1* polymorphism and the risk of cervical neoplasia with a borderline statistical significance. Larger sample size in this study may be responsible for the positive results. Our findings were in agreement with several previous reports. For example, Wang et al. reported that both *GSTM1* and *GSTT1* polymorphisms are associated with increased risk of hepatocellular carcinoma [77]. Economopoulos et al. reported that both *GSTM1* and *GSTT1* null genotype carriers exhibited higher colorectal cancer risk in Caucasian population [78]. In contrast, some researchers reported that *GSTM1* and *GSTT1* polymorphisms did not increase a substantial risk of breast cancer [88] and prostate cancer [102]. Taken together, these results indicate that *GSTM1* and *GSTT1* homozygous deletion polymorphisms may yield different effects on different types of cancers.

Our study has some limitations. Firstly, the between-study heterogeneity is a major problem in this meta-analysis because obvious heterogeneity was detected in overall analyses and also subgroup analyses. We explored several possible sources of heterogeneity, including cancer type, ethnicity, sample size, DNA source for genotyping and quality score. Unfortunately, we failed to find a bright reason for this variation, indicating that unknown confounding variables in single studies may have biased the findings. A conservative manner should, therefore, be adopted to interpret these results. Secondly, some potential confounding factors, such as age, sexual habits, and menopausal status can not be ruled out due to unadjusted data used. Finally, the sample size is relatively small in the meta-analysis, especially in some subgroup analyses.

In summary, this meta-analysis indicates that the null genotypes of *GSTM1* and *GSTT1* polymorphisms were associated with a significantly increased risk of cervical neoplasia. In *GSTM1*-*GSTT1* interaction analysis, individuals with dual null genotype were associated with a significantly increased risk of cervical neoplasia. In gene-environment interaction analysis, neither smoking status nor HPV infection status was associated with *GSTM1* and *GSTT1* polymorphisms. To ensure a precise estimate of the effect of *GSTM1* and *GSTT1* polymorphisms on cervical neoplasia risk, additional unbiased studies with larger sample size are needed. Such studies will not only elucidate the pivotal roles *GSTM1* and *GSTT1* polymorphisms playing in the development of cervical neoplasia, but also increase our understanding the etiology of cervical neoplasia.

## Supporting Information

Figure S1 Flow diagram of the literature search.
(TIF)

Figure S2 Forest plot of cervical neoplasia risk of *GSTM1* polymorphism in subgroup analysis according to ethnicity.
(TIF)

Figure S3 Forest plot of association between *GSTT1* polymorphism and risk of cervical neoplasia.
(TIF)

Figure S4 Forest plot of *GSTM1*-*GSTT1* interaction (null/null versus present/present).
(TIF)

Table S1 Quality assessment for the included studies.
(DOC)

Table S2 Overview of literatures included in the meta-analysis.
(DOC)

Table S3 Characteristics of the included studies.
(DOC)

Table S4 Summary odds ratios with confidence intervals between the *GSTM1* polymorphism and cervical neoplasia risk.
(DOC)

Table S5 Summary odds ratios with confidence intervals between the *GSTT1* polymorphism and cervical neoplasia risk.
(DOC)

Table S6 Summary odds ratios with confidence intervals between the *GSTM1-GSTT1* interaction and cervical neoplasia risk.
(DOC)

## References

[1] Parkin DM, Bray F, Ferlay J, Pisani P (2005). Global cancer statistics, 2002.. CA Cancer J Clin.

[2] Safaeian M, Solomon D, Castle PE (2007). Cervical cancer prevention–cervical screening: science in evolution.. Obstet Gynecol Clin North Am.

[3] Herrington CS (1999). Do HPV-negative cervical carcinomas exist?–revisited.. J Pathol.

[4] Walboomers JM, Meijer CJ (1997). Do HPV-negative cervical carcinomas exist?. J Pathol.

[5] Walboomers JM, Jacobs MV, Manos MM, Bosch FX, Kummer JA (1999). Human papillomavirus is a necessary cause of invasive cervical cancer worldwide.. J Pathol.

[6] Schiffman M, Castle PE, Jeronimo J, Rodriguez AC, Wacholder S (2007). Human papillomavirus and cervical cancer.. Lancet.

[7] Plummer M, Herrero R, Franceschi S, Meijer CJ, Snijders P (2003). Smoking and cervical cancer: pooled analysis of the IARC multi-centric case–control study.. Cancer Causes Control.

[8] Cancer. ICoESoC (2007). Comparison of risk factors for invasive squamous cell carcinoma and adenocarcinoma of the cervix: collaborative reanalysis of individual data on 8,097 women with squamous cell carcinoma and 1,374 women with adenocarcinoma from 12 epidemiological studies.. Int J Cancer.

[9] Appleby P, Beral V, Berrington de Gonzalez A, Colin D, Franceschi S (2007). Cervical cancer and hormonal contraceptives: collaborative reanalysis of individual data for 16,573 women with cervical cancer and 35,509 women without cervical cancer from 24 epidemiological studies.. Lancet.

[10] Jee SH, Lee JE, Park JS (2003). Polymorphism of codon 72 of p53 and environmental factors in the development of cervical cancer.. Int J Gynaecol Obstet.

[11] Magnusson PK, Sparen P, Gyllensten UB (1999). Genetic link to cervical tumours.. Nature.

[12] Hemminki K, Dong C, Vaittinen P (1999). Familial risks in cervical cancer: is there a hereditary component?. Int J Cancer.

[13] Magnusson PK, Gyllensten UB (2000). Cervical cancer risk: is there a genetic component?. Mol Med Today.

[14] Koushik A, Platt RW, Franco EL (2004). p53 codon 72 polymorphism and cervical neoplasia: a meta-analysis review.. Cancer Epidemiol Biomarkers Prev.

[15] Jee SH, Won SY, Yun JE, Lee JE, Park JS (2004). Polymorphism p53 codon-72 and invasive cervical cancer: a meta-analysis.. Int J Gynaecol Obstet.

[16] Sousa H, Santos AM, Pinto D, Medeiros R (2007). Is the p53 codon 72 polymorphism a key biomarker for cervical cancer development? A meta-analysis review within European populations.. Int J Mol Med.

[17] Klug SJ, Ressing M, Koenig J, Abba MC, Agorastos T (2009). TP53 codon 72 polymorphism and cervical cancer: a pooled analysis of individual data from 49 studies.. Lancet Oncol.

[18] McIlwain CC, Townsend DM, Tew KD (2006). Glutathione S-transferase polymorphisms: cancer incidence and therapy.. Oncogene.

[19] Hayes JD, Pulford DJ (1995). The glutathione S-transferase supergene family: regulation of GST and the contribution of the isoenzymes to cancer chemoprotection and drug resistance.. Crit Rev Biochem Mol Biol.

[20] Ketterer B (1988). Protective role of glutathione and glutathione transferases in mutagenesis and carcinogenesis.. Mutat Res.

[21] Strange RC, Spiteri MA, Ramachandran S, Fryer AA (2001). Glutathione-S-transferase family of enzymes.. Mutat Res.

[22] Pearson WR, Vorachek WR, Xu SJ, Berger R, Hart I (1993). Identification of class-mu glutathione transferase genes GSTM1-GSTM5 on human chromosome 1p13.. Am J Hum Genet.

[23] Webb G, Vaska V, Coggan M, Board P (1996). Chromosomal localization of the gene for the human theta class glutathione transferase (GSTT1).. Genomics.

[24] Hayes JD, Flanagan JU, Jowsey IR (2005). Glutathione transferases.. Annu Rev Pharmacol Toxicol.

[25] Warwick A, Sarhanis P, Redman C, Pemble S, Taylor JB (1994). Theta class glutathione S-transferase GSTT1 genotypes and susceptibility to cervical neoplasia: interactions with GSTM1, CYP2D6 and smoking.. Carcinogenesis.

[26] Warwick AP, Redman CW, Jones PW, Fryer AA, Gilford J (1994). Progression of cervical intraepithelial neoplasia to cervical cancer: interactions of cytochrome P450 CYP2D6 EM and glutathione s-transferase GSTM1 null genotypes and cigarette smoking.. Br J Cancer.

[27] de Carvalho CR, da Silva ID, Pereira JS, de Souza NC, Focchi GR (2008). Polymorphisms of p53, GSTM1 and GSTT1, and HPV in uterine cervix adenocarcinoma.. Eur J Gynaecol Oncol.

[28] Joseph T, Chacko P, Wesley R, Jayaprakash PG, James FV (2006). Germline genetic polymorphisms of CYP1A1, GSTM1 and GSTT1 genes in Indian cervical cancer: associations with tumor progression, age and human papillomavirus infection.. Gynecol Oncol.

[29] Sharma A, Sharma JK, Murthy NS, Mitra AB (2004). Polymorphisms at GSTM1 and GSTT1 gene loci and susceptibility to cervical cancer in Indian population.. Neoplasma.

[30] Singh H, Sachan R, Devi S, Pandey SN, Mittal B (2008). Association of GSTM1, GSTT1, and GSTM3 gene polymorphisms and susceptibility to cervical cancer in a North Indian population.. Am J Obstet Gynecol.

[31] Sobti RC, Kaur S, Kaur P, Singh J, Gupta I (2006). Interaction of passive smoking with GST (GSTM1, GSTT1, and GSTP1) genotypes in the risk of cervical cancer in India.. Cancer Genet Cytogenet.

[32] Palma S, Novelli F, Padua L, Venuti A, Prignano G (2010). Interaction between glutathione-S-transferase polymorphisms, smoking habit, and HPV infection in cervical cancer risk.. J Cancer Res Clin Oncol.

[33] Nishino K, Sekine M, Kodama S, Sudo N, Aoki Y (2008). Cigarette smoking and glutathione S-transferase M1 polymorphism associated with risk for uterine cervical cancer.. J Obstet Gynaecol Res.

[34] Niwa Y, Hirose K, Nakanishi T, Nawa A, Kuzuya K (2005). Association of the NAD(P)H: quinone oxidoreductase C609T polymorphism and the risk of cervical cancer in Japanese subjects.. Gynecol Oncol.

[35] Kim JW, Lee CG, Park YG, Kim KS, Kim IK (2000). Combined analysis of germline polymorphisms of p53, GSTM1, GSTT1, CYP1A1, and CYP2E1: relation to the incidence rate of cervical carcinoma.. Cancer.

[36] Lee SA, Kim JW, Roh JW, Choi JY, Lee KM (2004). Genetic polymorphisms of GSTM1, p21, p53 and HPV infection with cervical cancer in Korean women.. Gynecol Oncol.

[37] Settheetham-Ishida W, Yuenyao P, Kularbkaew C, Settheetham D, Ishida T (2009). Glutathione S-transferase (GSTM1 and GSTT1) polymorphisms in cervical cancer in Northeastern Thailand.. Asian Pac J Cancer Prev.

[38] Chen C, Madeleine MM, Weiss NS, Daling JR (1999). Glutathione S-transferase M1 genotypes and the risk of squamous carcinoma of the cervix: A population-based case-control study.. American Journal of Epidemiology.

[39] Sierra-Torres CH, Au WW, Arrastia CD, Cajas-Salazar N, Robazetti SC (2003). Polymorphisms for chemical metabolizing genes and risk for cervical neoplasia.. Environmental and Molecular Mutagenesis.

[40] Song GY, Shao SL, song ZY (2006). Association of single nucleotide polymorphism in glutathione S-transferase with susceptibility to cervical cancer.. TumourJournal of the World.

[41] Zhou Q, Wang J, Shao S, Ma X, Ding L (2006). The association between glutathione S-transferase M1, T1 polymorphisms and risk of cervical cancer. The study of the relationship between glutathione S-transferase M1, T1 genotypes and the risk of cervical cancer.. Modem Preventive Medicine.

[42] Ma C, Liu Y, Lu X, Ma Z (2009). Association between genetic polymorph ism of GSTM1 and susceptibility to cervical cancer in Uighur women in Xinjiang.. Chin J Obstet Gynecol.

[43] Huang YK, Hsieh HC, Sun JA, Chao CF, Huang RL (2006). Genetic polymorphisms of phase I and phase II xenobiotic enzymes in human papillomavirus related lesion and cancer of the uterine cervix.. Tzu Chi Medical Journal.

[44] Ueda M, Toji E, Nunobiki O, Izuma S, Okamoto Y (2008). Germline polymorphism of cancer susceptibility genes in gynecologic cancer.. Human Cell.

[45] Goodman MT, McDuffie K, Hernandez B, Bertram CC, Wilkens LR (2001). CYP1A1, GSTM1, and GSTT1 polymorphisms and the risk of cervical squamous intraepithelial lesions in a multiethnic population.. Gynecol Oncol.

[46] Agorastos T, Papadopoulos N, Lambropoulos AF, Chrisafi S, Mikos T (2007). Glutathione-S-transferase M1 and T1 and cytochrome P1A1 genetic polymorphisms and susceptibility to cervical intraepithelial neoplasia in Greek women.. Eur J Cancer Prev.

[47] Agodi A, Barchitta M, Cipresso R, Marzagalli R, La Rosa N (2010). Distribution of p53, GST, and MTHFR polymorphisms and risk of cervical intraepithelial lesions in sicily.. International Journal of Gynecological Cancer.

[48] Ueda M, Hung YC, Terai Y, Saito J, Nunobiki O (2005). Glutathione-S-transferase and p53 polymorphisms in cervical carcinogenesis.. Gynecologic Oncology.

[49] Sierra-Torres CH, Arboleda-Moreno YY, Orejuela-Aristizabal L (2006). Exposure to wood smoke, HPV infection, and genetic susceptibility for cervical neoplasia among women in Colombia.. Environ Mol Mutagen.

[50] Liu Y, Ma W, Liu Q, Yang T, Xu X (2009). Association between genetic polymorph ism of GSTM1, CYP2E1 and susceptibility to cervical cancer and its precancerous lesions in Uighur women in Xinjiang.. Prog Obstet Gynecol.

[51] Song GY, Song ZY, Xu JP, Shao SL (2008). Association of single nucleotide polymorphism in glutathione S-transferase-M1 with susceptibility to cervical cancer in Shanxi Province.. Chinese Journal of Cancer Prevention and Treatment.

[52] Camargo MC, Mera R, Correa P, Peek RM, Fontham ET (2006). Interleukin-1beta and interleukin-1 receptor antagonist gene polymorphisms and gastric cancer: a meta-analysis.. Cancer Epidemiol Biomarkers Prev.

[53] Thakkinstian A, McEvoy M, Minelli C, Gibson P, Hancox B (2005). Systematic review and meta-analysis of the association between {beta}2-adrenoceptor polymorphisms and asthma: a HuGE review.. Am J Epidemiol.

[54] Gao LB, Pan XM, Li LJ, Liang WB, Zhu Y (2010). RAD51 135G/C polymorphism and breast cancer risk: a meta-analysis from 21 studies.. Breast Cancer Res Treat [Epub ahead of print].

[55] Higgins JP, Thompson SG (2002). Quantifying heterogeneity in a meta-analysis.. Stat Med.

[56] Higgins JP, Thompson SG, Deeks JJ, Altman DG (2003). Measuring inconsistency in meta-analyses.. BMJ.

[57] Mantel N, Haenszel W (1959). Statistical aspects of the analysis of data from retrospective studies of disease.. J Natl Cancer Inst.

[58] DerSimonian R, Laird N (1986). Meta-analysis in clinical trials.. Control Clin Trials.

[59] Egger M, Davey Smith G, Schneider M, Minder C (1997). Bias in meta-analysis detected by a simple, graphical test.. BMJ.

[60] Norppa H (2004). Cytogenetic biomarkers and genetic polymorphisms.. Toxicol Lett.

[61] Hayes JD, Strange RC (2000). Glutathione S-transferase polymorphisms and their biological consequences.. Pharmacology.

[62] Garte S, Gaspari L, Alexandrie AK, Ambrosone C, Autrup H (2001). Metabolic gene polymorphism frequencies in control populations.. Cancer Epidemiol Biomarkers Prev.

[63] Winkelstein W (1977). Smoking and cancer of the uterine cervix: hypothesis.. Am J Epidemiol.

[64] Winkelstein W, Shillitoe EJ, Brand R, Johnson KK (1984). Further comments on cancer of the uterine cervix, smoking, and herpesvirus infection.. Am J Epidemiol.

[65] Winkelstein W (1990). Smoking and cervical cancer–current status: a review.. Am J Epidemiol.

[66] Steckley SL, Pickworth WB, Haverkos HW (2003). Cigarette smoking and cervical cancer: Part II: a geographic variability study.. Biomed Pharmacother.

[67] Haverkos HW, Soon G, Steckley SL, Pickworth W (2003). Cigarette smoking and cervical cancer: Part I: a meta-analysis.. Biomed Pharmacother.

[68] Greenberg ER, Vessey M, McPherson K, Yeates D (1985). Cigarette smoking and cancer of the uterine cervix.. Br J Cancer.

[69] Sood AK (1991). Cigarette smoking and cervical cancer: meta-analysis and critical review of recent studies.. Am J Prev Med.

[70] Prokopczyk B, Cox JE, Hoffmann D, Waggoner SE (1997). Identification of tobacco-specific carcinogen in the cervical mucus of smokers and nonsmokers.. J Natl Cancer Inst.

[71] Hoffmann D, Hoffmann I (1997). The changing cigarette, 1950–1995.. J Toxicol Environ Health.

[72] Kjellberg L, Hallmans G, Ahren AM, Johansson R, Bergman F (2000). Smoking, diet, pregnancy and oral contraceptive use as risk factors for cervical intra-epithelial neoplasia in relation to human papillomavirus infection.. Br J Cancer.

[73] Kuper H, Adami HO, Boffetta P (2002). Tobacco use, cancer causation and public health impact.. J Intern Med.

[74] Nadais Rda F, Campaner AB, Piato S, Longo Galvao MA, dos Santos RE (2006). Langerhans' cells and smoking in intraepithelial neoplasia of the cervix.. Gynecol Oncol.

[75] Lai R, Crevier L, Thabane L (2005). Genetic polymorphisms of glutathione S-transferases and the risk of adult brain tumors: a meta-analysis.. Cancer Epidemiol Biomarkers Prev.

[76] White DL, Li D, Nurgalieva Z, El-Serag HB (2008). Genetic variants of glutathione S-transferase as possible risk factors for hepatocellular carcinoma: a HuGE systematic review and meta-analysis.. Am J Epidemiol.

[77] Wang B, Huang G, Wang D, Li A, Xu Z (2010). Null genotypes of GSTM1 and GSTT1 contribute to hepatocellular carcinoma risk: evidence from an updated meta-analysis.. J Hepatol.

[78] Economopoulos KP, Sergentanis TN (2010). GSTM1, GSTT1, GSTP1, GSTA1 and colorectal cancer risk: a comprehensive meta-analysis.. Eur J Cancer.

[79] Raimondi S, Botteri E, Iodice S, Lowenfels AB, Maisonneuve P (2009). Gene-smoking interaction on colorectal adenoma and cancer risk: review and meta-analysis.. Mutat Res.

[80] Saadat M (2006). Genetic polymorphisms of glutathione S-transferase T1 (GSTT1) and susceptibility to gastric cancer: a meta-analysis.. Cancer Sci.

[81] Chen B, Cao L, Zhou Y, Yang P, Wan HW (2010). Glutathione S-transferase T1 (GSTT1) gene polymorphism and gastric cancer susceptibility: a meta-analysis of epidemiologic studies.. Dig Dis Sci.

[82] Boccia S, La Torre G, Gianfagna F, Mannocci A, Ricciardi G (2006). Glutathione S-transferase T1 status and gastric cancer risk: a meta-analysis of the literature.. Mutagenesis.

[83] La Torre G, Boccia S, Ricciardi G (2005). Glutathione S-transferase M1 status and gastric cancer risk: a meta-analysis.. Cancer Lett.

[84] Wang H, Zhou Y, Zhuang W, Yin YQ, Liu GJ (2010). Glutathione S-transferase M1 null genotype associated with gastric cancer among Asians.. Dig Dis Sci.

[85] Sergentanis TN, Economopoulos KP (2010). GSTT1 and GSTP1 polymorphisms and breast cancer risk: a meta-analysis.. Breast Cancer Res Treat.

[86] Sull JW, Ohrr H, Kang DR, Nam CM (2004). Glutathione S-transferase M1 status and breast cancer risk: a meta-analysis.. Yonsei Med J.

[87] Qiu LX, Yuan H, Yu KD, Mao C, Chen B (2010). Glutathione S-transferase M1 polymorphism and breast cancer susceptibility: a meta-analysis involving 46,281 subjects.. Breast Cancer Res Treat.

[88] Vogl FD, Taioli E, Maugard C, Zheng W, Pinto LF (2004). Glutathione S-transferases M1, T1, and P1 and breast cancer: a pooled analysis.. Cancer Epidemiol Biomarkers Prev.

[89] Egan KM, Cai Q, Shu XO, Jin F, Zhu TL (2004). Genetic polymorphisms in GSTM1, GSTP1, and GSTT1 and the risk for breast cancer: results from the Shanghai Breast Cancer Study and meta-analysis.. Cancer Epidemiol Biomarkers Prev.

[90] Gao Y, Cao Y, Tan A, Liao C, Mo Z (2010). Glutathione S-transferase M1 polymorphism and sporadic colorectal cancer risk: An updating meta-analysis and HuGE review of 36 case-control studies.. Ann Epidemiol.

[91] Johns LE, Houlston RS (2000). Glutathione S-transferase mu1 (GSTM1) status and bladder cancer risk: a meta-analysis.. Mutagenesis.

[92] Garcia-Closas M, Malats N, Silverman D, Dosemeci M, Kogevinas M (2005). NAT2 slow acetylation, GSTM1 null genotype, and risk of bladder cancer: results from the Spanish Bladder Cancer Study and meta-analyses.. Lancet.

[93] Engel LS, Taioli E, Pfeiffer R, Garcia-Closas M, Marcus PM (2002). Pooled analysis and meta-analysis of glutathione S-transferase M1 and bladder cancer: a HuGE review.. Am J Epidemiol.

[94] Benhamou S, Lee WJ, Alexandrie AK, Boffetta P, Bouchardy C (2002). Meta- and pooled analyses of the effects of glutathione S-transferase M1 polymorphisms and smoking on lung cancer risk.. Carcinogenesis.

[95] Shi X, Zhou S, Wang Z, Zhou Z (2008). CYP1A1 and GSTM1 polymorphisms and lung cancer risk in Chinese populations: a meta-analysis.. Lung Cancer.

[96] McWilliams JE, Sanderson BJ, Harris EL, Richert-Boe KE, Henner WD (1995). Glutathione S-transferase M1 (GSTM1) deficiency and lung cancer risk.. Cancer Epidemiol Biomarkers Prev.

[97] Houlston RS (1999). Glutathione S-transferase M1 status and lung cancer risk: a meta-analysis.. Cancer Epidemiol Biomarkers Prev.

[98] Carlsten C, Sagoo GS, Frodsham AJ, Burke W, Higgins JP (2008). Glutathione S-transferase M1 (GSTM1) polymorphisms and lung cancer: a literature-based systematic HuGE review and meta-analysis.. Am J Epidemiol.

[99] Ye Z, Song H, Higgins JP, Pharoah P, Danesh J (2006). Five glutathione s-transferase gene variants in 23,452 cases of lung cancer and 30,397 controls: meta-analysis of 130 studies.. PLoS Med.

[100] Zhuo WL, Zhang YS, Wang Y, Zhuo XL, Zhu B (2009). Association studies of CYP1A1 and GSTM1 polymorphisms with esophageal cancer risk: evidence-based meta-analyses.. Arch Med Res.

[101] Yang CX, Matsuo K, Wang ZM, Tajima K (2005). Phase I/II enzyme gene polymorphisms and esophageal cancer risk: a meta-analysis of the literature.. World J Gastroenterol.

[102] Ntais C, Polycarpou A, Ioannidis JP (2005). Association of GSTM1, GSTT1, and GSTP1 gene polymorphisms with the risk of prostate cancer: a meta-analysis.. Cancer Epidemiol Biomarkers Prev.

[103] Mo Z, Gao Y, Cao Y, Gao F, Jian L (2009). An updating meta-analysis of the GSTM1, GSTT1, and GSTP1 polymorphisms and prostate cancer: a HuGE review.. Prostate.

[104] Zhuo X, Cai L, Xiang Z, Li Q, Zhang X (2009). GSTM1 and GSTT1 polymorphisms and nasopharyngeal cancer risk: an evidence-based meta-analysis.. J Exp Clin Cancer Res.

[105] Hashibe M, Brennan P, Strange RC, Bhisey R, Cascorbi I (2003). Meta- and pooled analyses of GSTM1, GSTT1, GSTP1, and CYP1A1 genotypes and risk of head and neck cancer.. Cancer Epidemiol Biomarkers Prev.

[106] Zhuo W, Wang Y, Zhuo X, Zhu Y, Wang W (2009). CYP1A1 and GSTM1 polymorphisms and oral cancer risk: association studies via evidence-based meta-analyses.. Cancer Invest.

[107] Varela-Lema L, Taioli E, Ruano-Ravina A, Barros-Dios JM, Anantharaman D (2008). Meta-analysis and pooled analysis of GSTM1 and CYP1A1 polymorphisms and oral and pharyngeal cancers: a HuGE-GSEC review.. Genet Med.

[108] Zhuo WL, Wang Y, Zhuo XL, Zhu B, Zhu Y (2009). Polymorphisms of CYP1A1 and GSTM1 and laryngeal cancer risk: evidence-based meta-analyses.. J Cancer Res Clin Oncol.

[109] Das P, Shaik AP, Bammidi VK (2009). Meta-analysis study of glutathione-S-transferases (GSTM1, GSTP1, and GSTT1) gene polymorphisms and risk of acute myeloid leukemia.. Leuk Lymphoma.

[110] Ye Z, Song H (2005). Glutathione s-transferase polymorphisms (GSTM1, GSTP1 and GSTT1) and the risk of acute leukaemia: a systematic review and meta-analysis.. Eur J Cancer.

[111] Economopoulos KP, Choussein S, Vlahos NF, Sergentanis TN (2010). GSTM1 polymorphism, GSTT1 polymorphism, and cervical cancer risk: a meta-analysis.. Int J Gynecol Cancer.

