# Supplementary figures and images for: Null Genotypes of GSTM1 and GSTT1 Contribute to Risk of Cervical Neoplasia: An Evidence-Based Meta-Analysis
Source: PLoS One. 2011 May 23;6(5):e20157. doi: 10.1371/journal.pone.0020157 (PMC3100325; doi:10.1371/journal.pone.0020157)

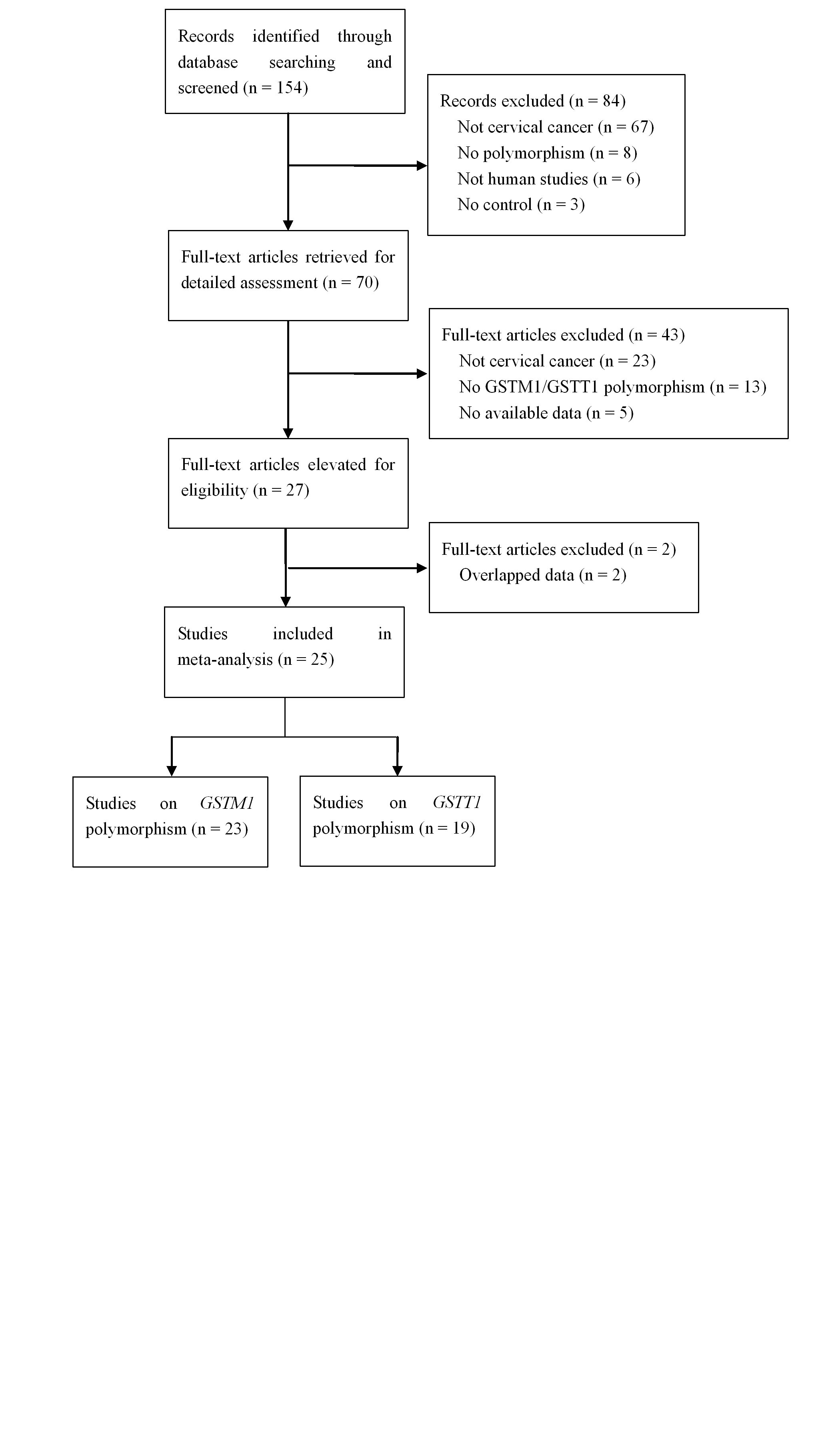

Supplement: Figure S1 — Flow diagram of the literature search. (TIF) [file pone.0020157.s001.tif]

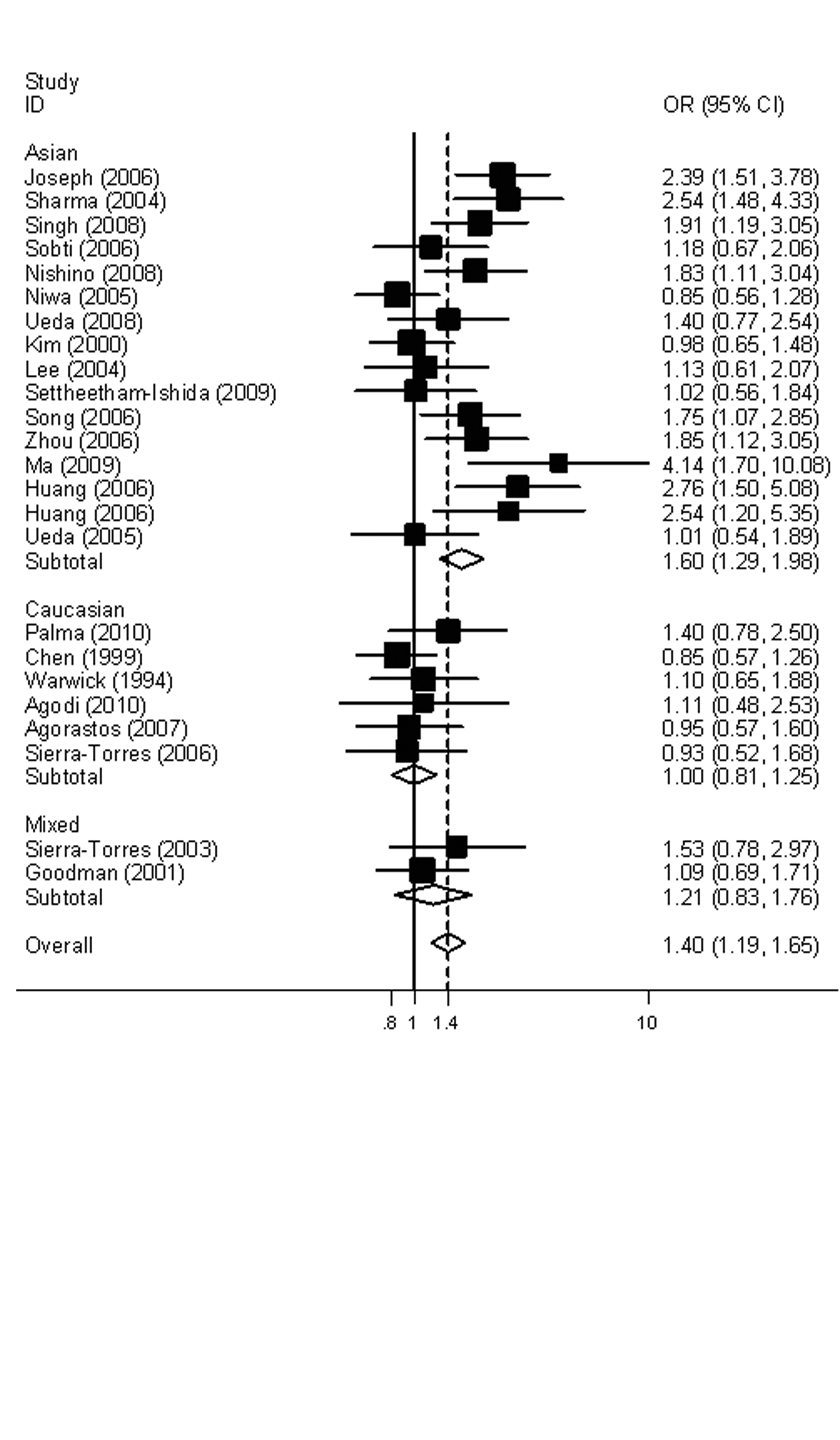

Supplement: Figure S2 — Forest plot of cervical neoplasia risk of GSTM1 polymorphism in subgroup analysis according to ethnicity. (TIF) [file pone.0020157.s002.tif]

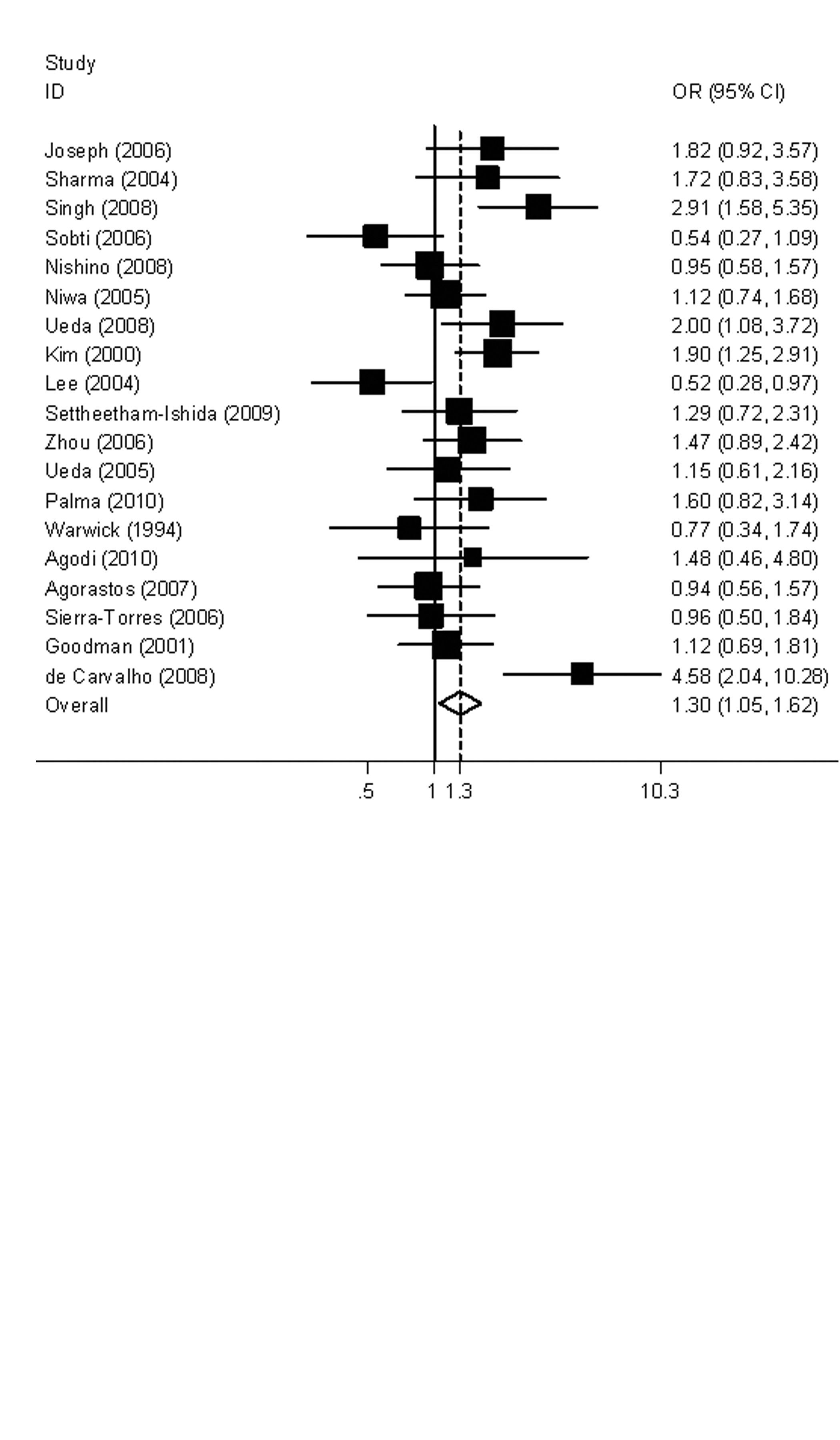

Supplement: Figure S3 — Forest plot of association between GSTT1 polymorphism and risk of cervical neoplasia. (TIF) [file pone.0020157.s003.tif]

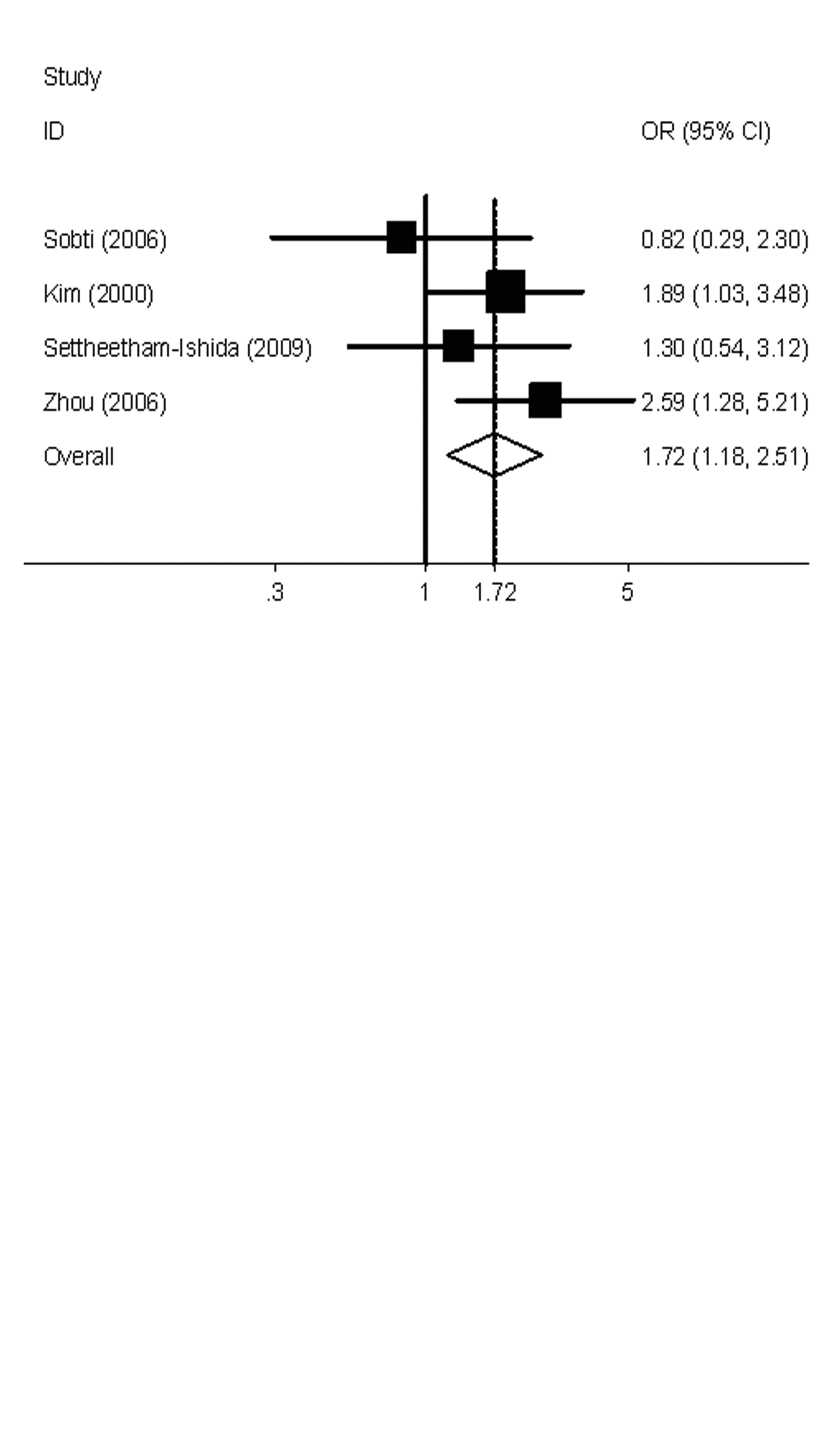

Supplement: Figure S4 — Forest plot of GSTM1-GSTT1 interaction (null/null versus present/present). (TIF) [file pone.0020157.s004.tif]
