# Supplementary material for: Null Genotypes of GSTM1 and GSTT1 Contribute to Risk of Cervical Neoplasia: An Evidence-Based Meta-Analysis
Source: PLoS One. 2011 May 23;6(5):e20157. doi: 10.1371/journal.pone.0020157 (PMC3100325; doi:10.1371/journal.pone.0020157)
Supplement: Table S1 — Quality assessment for the included studies. (DOC) [file pone.0020157.s005.doc]

| **Quality parameters** | **Score** |
| --- | --- |
| **Source of cases** |  |
| Selected from cancer registry | 2 |
| Selected from hospital or cancer institute | 1 |
| Not described | 0 |
| **Source of controls** |  |
| Population-based | 2 |
| Healthy volunteers/blood donors with complete description | 1.5 |
| Hospital-based controls without cervical lesions | 1 |
| Healthy volunteers/blood donors without complete description | 0.5 |
| Patients with cervical lesions or other diseases | 0.25 |
| Not described | 0 |
| **Ascertainment of cervical cancer** |  |
| Cytological and/or colposcopical and/or histological confirmation | 2 |
| Patient medical record | 1 |
| Not described | 0 |
| **Sample size (total number of cases and controls)** |  |
| > 200 | 2 |
| 100-200 | 1 |
| < 100 | 0 |
| **Quality control of genotyping methods** |  |
| Clearly described an internal control and repetition to confirm the data | 2 |
| An internal control was used to confirm the data | 1 |
| Not described | 0 |
| **Matching of case and control participants** |  |
| More than one variable (such as age and ethnicity) | 2 |
| Only one variable (such as age or ethnicity) | 1 |
| Not described | 0 |
