# Supplementary material for: Null Genotypes of GSTM1 and GSTT1 Contribute to Risk of Cervical Neoplasia: An Evidence-Based Meta-Analysis
Source: PLoS One. 2011 May 23;6(5):e20157. doi: 10.1371/journal.pone.0020157 (PMC3100325; doi:10.1371/journal.pone.0020157)
Supplement: Table S2 — Overview of literatures included in the meta-analysis. (DOC) [file pone.0020157.s006.doc]

| Study (first author) | Year of publication | Country (region) | Year of sample collection | Ethnicity | Source and ascertainment of  cases | Type of controls | Sample size (cases/ controls) | Source of DNA for genotyping | Matching criteria | Polymorphisms evaluated |
| --- | --- | --- | --- | --- | --- | --- | --- | --- | --- | --- |
| de Carvalho | 2008 | Brazil | NA | NA | Tumor fragments obtained from patients with adenocarcinoma and confirmed by histopathologic examination and without treatment | Endocervical scraping without intraepithelial and inferior genital tract invading neoplasia | 43/86 | Tumor tissue (cases); exfoliated cervical cells (controls) | - | GSTT1 |
| Joseph | 2006 | India (Thiruvananthapuram) | NA | Asian | Patients with invasive cervical cancer or those with H-SILs were enrolled from Regional Cancer Centre | Normal healthy women and subjects diagnosed with benign cytology and L-SILs | 147/165 | Cervical biopsies exfoliated cervical cells and white blood cells | Age, marital status, parity, monthly income, religion and socioeconomic status | GSTM1, GSTT1 |
| Sharma | 2004 | India (New Delhi) | 1994 | Asian | Peripheral blood samples from patients with squamous cell carcinoma were recruited from the cancer clinic of Lok Nayak Hospital | Normal volunteers without any history of chronic disease | 142/96 | White blood cells | - | GSTM1, GSTT1 |
| Singh | 2008 | India (Uttar Pradesh) | 2005-2006 | Asian | Cervical cancer cases were taken from Queen Mary Hospital and confirmed by cervical biopsy | Healthy blood donors without cervical dysplasia | 150/168 | White blood cells | - | GSTM1, GSTT1 |
| Sobti | 2006 | India (Ludhiana (Punjab)) | NA | Asian | Histologically confirmed cervix cancer patients were collected from Post Graduate Institute of Medical Education and Research and Mohan Dai Oswal Cancer Hospital | Individuals without any kind of malignancy | 103/103 | White blood cells | Age | GSTM1, GSTT1 |
| Palma | 2010 | Italy (Rome) | 2006-2007 | Caucasian | The cytological specimens were collected from San Gallicano Hospital | Healthy controls without any evidence of cervical lesions | 81/111 | Exfoliated cervical cells | Age | GSTM1, GSTT1 |
| Nishino | 2008 | Japan (Niigata) | NA | Asian | 124 patients with CIN3 or invasive uterine cervical squamous cell cancer without a history of treatment were enrolled and all the diagnoses were confirmed by surgical specimens | Healthy controls without any evidence of cervical lesions | 124/125 | White blood cells | Age | GSTM1, GSTT1 |
| Niwa | 2005 | Japan (Nagoya) | 2001-2003 | Asian | 131 cervical cancer patients who were histologically diagnosed were enrolled from Aichi Cancer Center Hospital | Controls without a history of cancer | 131/320 | White blood cells | - | GSTM1, GSTT1 |
| Ueda | 2008 | Japan (Osaka) | NA | Asian | Blood samples | Healthy blood donors | 83/95 | White blood cells | - | GSTM1, GSTT1 |
| Kim | 2000 | Korea (Seoul) | 1995-1997 | Asian | 181 histologically confirmed oncogenic HPV-16 or HPV-18 positive cervical carcinoma patients were selected | Laboratory personnel, students, and other female volunteers | 181/181 | White blood cells | Age | GSTM1, GSTT1 |
| Lee | 2004 | Korea (Seoul) | 1997-1999 | Asian | From 1997 to 1999, uterine cervical carcinoma was diagnosed at the Department of Obstetrics and Gynecology of Seoul National University Hospital | The controls had no evidence of cervical lesions | 81/86 | White blood cells | - | GSTM1, GSTT1 |
| Settheetham-Ishida | 2009 | Thailand (Khon Kaen) | NA | Asian | 90 patients with squamous cell cervical cancer were confirmed by cytological, colposcopical and histological diagnosis | Healthy women free from cervical cancer, history of conization, hysteretomy or diseases which are associated with known risk factors for cervical cancer | 90/94 | White blood cells | Age | GSTM1, GSTT1 |
| Chen | 1999 | America (Seattle) | 1987-1995 | Caucasian | 190 patients with squamous cell cervical cancer were enrolled from a population-based registry and confirmed by histologic biopsy | 206 controls without a history of cervical cancer or hysterectomy were identified using random-digit telephone dialing | 190/206 | White blood cells | Age | GSTM1 |
| Song | 2006 | China (Shanxi) | 2005-2006 | Asian | Patients were confirmed by histopathologic examination | Healthy controls | 130/130 | White blood cells | Age | GSTM1 |
| Zhou | 2006 | China (Shanxi) | 2004 | Asian | Patients were confirmed by histopathologic examination | Patients with hysteromyoma were defined as controls | 125/125 | White blood cells | Age | GSTM1, GSTT1 |
| Ma | 2009 | China (Xinjiang) | 2000-2005 | Asian | ICC cases without other tumors and chemoradiation were enrolled | Patients without ICC and CIN were defined as controls | 43/45 | Tissue sample | Age | GSTM1 |
| Sierra-Torres | 2003 | America (Texas) | NA | Mixed (49% Caucasians; 37% hispanics; 14% African Americans) | Women with HGCIN (n=39) and ICC (n=37) confirmed by biopsy were selected | Women with a history of normal Pap tests (> 1 year) were defined as controls | 69/72 | White blood cells | Age (± 5 years) and ethnicity | GSTM1 |
| Huang | 2006 | China (Taiwan) | 1993-2000 | Asian | L-SILs and ICC cases were diagnosed by a committee of staff members, including colposcoposts, cytologists and pathologists | Controls were defined as women without a history of cervical neoplasia, HPV-related diseases such as skin or genital warts, immune-compromised conditions or chronic or acute cervicitis | 80/80 (L-SIL/control); 113/113 (ICC/control) | Tumor tissue (ICC cases); exfoliated cervical cells (L-SILs cases and controls) | Age (within 3 years) | GSTM1 |
| Warwick | 1994a | UK (Staffordshire) | NA | Caucasian | 77 squamous cell cervical cancer patients who presented *de novo* or were under surveillance following completion of treatment were enrolled | 190 unrelated Caucasians (70% female) without clinical or histological evidence of malignant or inflammatory disease were defined as controls | 77/190 | NA | - | GSTM1 |
| Warwick | 1994b | UK (Staffordshire) | NA | Caucasian | Squamous cell cervical cancer patients who presented *de novo* or were under surveillance following completion of treatment were enrolled | Women with normal cervical pathology suffering menorrhagia were defined as controls | 70/168 | NA | - | GSTT1 |
| Agodi | 2010 | Italy (Catania) | NA | Caucasian | Patients with CIN 2 or CIN 3 were enrolled | Women with no cervical abnormalities or with CIN 1 were defined as controls | 27/162 | Exfoliated cervical cells | - | GSTM1, GSTT1 |
| Agorastos | 2007 | Greece (Thessaloniki) | 1999-2003 | Caucasian | 176 patients with cervical neoplasia of any degree were confirmed histologically and enrolled | Women without cytological or colposcopical evidence of cervical pathology were used as control | 176/114 | NA | - | GSTM1, GSTT1 |
| Goodman | 2001 | America (*Hawaii*) | 1992-1996 | Mixed (128 Caucasians; 45 Hawaiians; 47 Japaneses;91 others) | Women with biopsy-confirmed SIL were enrolled | Women with negative cytological results were selected as control | 131/180 | White blood cells | - | GSTM1, GSTT1 |
| Ueda | 2005 | Japan (Osaka) | NA | Asian | Women with L-SILs (n=102) and H-SILs (n=42) confirmed by colposcopy-directed biopsy were selected | Nomal Japanese women | 144/54 | Exfoliated cervical cells | - | GSTM1, GSTT1 |
| Sierra-Torres | 2006 | America (Colombia) | 1993 | Caucasian | Women with histologically confirmed H-SILs were defined as cases | Women with a history of normal Pap tests (≥1 year) were defined as controls | 91/92 | White blood cells | Age and place of origin (urban or rural) | GSTM1, GSTT1 |

NA, not available. H-SILs, high-grade squamous intraepithelial lesions. L-SILs, low-grade squamous intraepithelial lesions. CIN, cervical intraepithelial neoplasia. HPV, human papilloma virus. HGCIN, high-grade cervical intraepithelial neoplasia. ICC, invasive cervical cancer.
