# Supplementary material for: Null Genotypes of GSTM1 and GSTT1 Contribute to Risk of Cervical Neoplasia: An Evidence-Based Meta-Analysis
Source: PLoS One. 2011 May 23;6(5):e20157. doi: 10.1371/journal.pone.0020157 (PMC3100325; doi:10.1371/journal.pone.0020157)
Supplement: Table S3 — Characteristics of the included studies. (DOC) [file pone.0020157.s007.doc]

|  |  | *GSTM1* polymorphism | |  | *GSTT1* polymorphism | |
| --- | --- | --- | --- | --- | --- | --- |
|  |  | Case (%) | Control (%) |  | Case (%) | Control (%) |
| Pathologic types |  |  |  |  |  |  |
| Cervical cancer (unclear type) |  | 851 (32.5) | - |  | 566 (27.1) | - |
| SCC |  | 836 (31.9) | - |  | 639 (30.5) | - |
| HGL |  | 371 (14.2) | - |  | 332 (15.9) | - |
| LGL |  | 226 (8.6) | 75 (2.4) |  | 179 (8.6) | 75 (3.0) |
| SIL (unknown grade) |  | 131 (5.0) | - |  | 131 (6.3) | - |
| Mixed A |  | 124 (4.7) | - |  | 124 (5.9) | - |
| AC |  | 78 (3.0) | - |  | 121 (5.8) | - |
| Mixed B |  | - | 162 (5.3) |  | - | 162 (6.5) |
| Hysteromyoma and/or unknown |  | - | 170 (5.5) |  | - | 125 (5.0) |
| Normal |  | - | 2,678 (86.8) |  | - | 2,142 (85.5) |
| Ethnicities |  |  |  |  |  |  |
| Asian |  | 1,811 (69.2) | 2,004 (64.9) |  | 1,516 (72.5) | 1,638 (65.4) |
| Caucasian |  | 704 (26.9) | 970 (31.4) |  | 470 (22.5) | 707 (28.2) |
| Hispanic |  | 28 (1.1) | 25 (0.8) |  | - | - |
| Hawaiian |  | 19 (0.7) | 26 (0.8) |  | 19 (0.9) | 26 (1.0) |
| African American |  | 11 (0.4) | 15 (0.5) |  | - | - |
| other |  | 44 (1.7) | 47 (1.5) |  | 87 (4.2) | 133 (5.3) |
| Smoking status |  |  |  |  |  |  |
| Smoking |  | 564 (21.6) | 410 (13.3) |  | 414 (19.8) | 353 (14.1) |
| Non-smoking |  | 491 (18.8) | 869 (28.2) |  | 443 (21.2) | 813 (32.5) |
| No data |  | 1,555 (59.6) | 1,805 (58.5) |  | 1,235 (59.0) | 1,338 (53.4) |
| HPV infection status |  |  |  |  |  |  |
| HPV positive |  | 678 (26.0) | 171 (5.5) |  | 480 (22.9) | 167 (6.7) |
| HPV negative |  | 291 (11.1) | 821 (26.6) |  | 224 (10.7) | 607 (24.2) |
| No data |  | 1,641 (62.9) | 2,092 (67.8) |  | 1,388 (66.3) | 1,730 (69.1) |

SCC, squamous cell carcinoma. HGL, high-grade squamous intraepithelial lesions and cervical intraepithelial lesions grades 2 and 3. LGL, low-grade squamous intraepithelial lesions and cervical intraepithelial lesions grade 1. SIL, squamous intraepithelial lesions. Mixed A, invasive cervical cancer and high-grade lesions. AC, adenocarcinoma and adenosquamous carcinoma. Mixed B, normal control and CIN1. HPV, human papilloma virus.
