# Supplementary material for: Null Genotypes of GSTM1 and GSTT1 Contribute to Risk of Cervical Neoplasia: An Evidence-Based Meta-Analysis
Source: PLoS One. 2011 May 23;6(5):e20157. doi: 10.1371/journal.pone.0020157 (PMC3100325; doi:10.1371/journal.pone.0020157)
Supplement: Table S4 — Summary odds ratios with confidence intervals between the GSTM1 polymorphism and cervical neoplasia risk. (DOC) [file pone.0020157.s008.doc]

| *GSTM1* | n ^a^ | Cases/  controls | Heterogeneity | |  | Model for  meta-analysis ^c^ |  | Null versus present | |  | *P* _Egger’s test_ ^d^ |
| --- | --- | --- | --- | --- | --- | --- | --- | --- | --- | --- | --- |
|  |  |  | *I*^2^ (%) | *P* _heterogeneity_ ^b^ |  |  |  | OR (95%CI) | *P* |  |  |
| Total | 23 | 2,610/3,084 | 53.3 | 0.001 |  | R |  | 1.40 (1.19-1.65) | <0.001 |  | 0.087 |
| Pathologic types |  |  |  |  |  |  |  |  |  |  |  |
| Cervical cancer (unclear type) | 10 | 1,024/1,368 | 61.2 | 0.006 |  | R |  | 1.54 (1.16-2.04) | 0.003 |  | 0.082 |
| SCC ^e^ | 5 | 591/689 | 63.0 | 0.029 |  | R |  | 1.21 (0.83-1.78) | 0.325 |  | 0.433 |
| HGL ^f^ | 5 | 287/518 | 0.0 | 0.783 |  | F |  | 0.99 (0.73-1.34) | 0.934 |  | 0.262 |
| LGL ^g^ | 4 | 226/342 | 8.3 | 0.352 |  | F |  | 1.40 (0.98-2.02) | 0.068 |  | 0.737 |
| Mixed ^h^ | 3 | 340/362 | 0.0 | 0.518 |  | F |  | 1.98 (1.46-2.68) | <0.001 |  | 0.295 |
| AC ^i^ | 1 | 11/103 | - | - |  | R/F |  | 1.43 (0.41-4.99) | 0.579 |  | - |
| Ethnicities |  |  |  |  |  |  |  |  |  |  |  |
| Asian | 16 | 1,796/1,972 | 59.8 | 0.001 |  | R |  | 1.60 (1.29-1.98) | <0.001 |  | 0.101 |
| Caucasian | 6 | 614/860 | 0.0 | 0.814 |  | F |  | 1.00 (0.81-1.25) | 0.979 |  | 0.230 |
| Mixed ^j^ | 2 | 200/252 | 0.0 | 0.408 |  | F |  | 1.21 (0.83-1.76) | 0.319 |  | - |
| Source of DNA for genotyping |  |  |  |  |  |  |  |  |  |  |  |
| White blood cells | 15 | 1,821/2,069 | 48.2 | 0.019 |  | R |  | 1.29 (1.08-1.55) | 0.006 |  | 0.169 |
| Exfoliated cervical cells | 4 | 299/405 | 20.8 | 0.285 |  | F |  | 1.39 (0.99-1.94) | 0.057 |  | 0.764 |
| Tissue sample | 2 | 118/156 | 0.0 | 0.462 |  | F |  | 3.14 (1.90-5.19) | <0.001 |  | - |
| No available data | 2 | 225/289 | 0.0 | 0.698 |  | F |  | 1.02 (0.71-1.48) | 0.898 |  | - |
| Mixed ^k^ | 1 | 147/165 | - | - |  | R/F |  | 2.39 (1.51-3.78) | <0.001 |  | - |
| Quality criteria |  |  |  |  |  |  |  |  |  |  |  |
| Quality score ≥ 7 | 10 | 1,253/1,292 | 44.3 | 0.064 |  | R |  | 1.31 (1.06-1.62) | 0.014 |  | 0.463 |
| Quality score < 7 | 14 | 1,357/1,792 | 62.3 | 0.001 |  | R |  | 1.49 (1.16-1.91) | 0.002 |  | 0.145 |
| Matched (age) | 14 | 1,496/1,638 | 61.0 | 0.002 |  | R |  | 1.54 (1.22-1.95) | <0.001 |  | 0.053 |
| Unmatched (age) | 10 | 1,114/1,446 | 42.3 | 0.076 |  | R |  | 1.24 (0.99-1.55) | 0.066 |  | 0.746 |
| Smoking status |  |  |  |  |  |  |  |  |  |  |  |
| Smoking | 6 | 414/287 | 0.0 | 0.654 |  | F |  | 1.04 (0.76-1.41) | 0.823 |  | 0.054 |
| Non-smoking | 6 | 323/417 | 35.4 | 0.171 |  | F |  | 1.06 (0.78-1.43) | 0.717 |  | 0.969 |
| HPV infection status |  |  |  |  |  |  |  |  |  |  |  |
| HPV positive | 4 | 254/110 | 59.8 | 0.058 |  | R |  | 1.59 (0.71-3.55) | 0.257 |  | 0.750 |
| HPV negative | 3 | 100/222 | 71.2 | 0.031 |  | R |  | 1.67 (0.60-4.66) | 0.330 |  | 0.634 |
| Age (years) |  |  |  |  |  |  |  |  |  |  |  |
| > 40 | 2 | 145/150 | 6.5 | 0.301 |  | F |  | 0.73 (0.46-1.15) | 0.178 |  | - |
| ≤ 40 | 2 | 166/161 | 0.0 | 0.564 |  | F |  | 2.02 (1.30-3.14) | 0.002 |  | - |

^a^ Number of studies. ^b^ *P* _heterogeneity_, *P* value of Q-test for heterogeneity test. ^c^ R, random-effects model; F, fixed-effects model. ^d^ *P* _Egger’s test_, the *P* value for Egger’s test. ^e^ SCC, squamous cell carcinoma. ^f^ HGL, high-grade squamous intraepithelial lesions and cervical intraepithelial lesions grades 2 and 3. ^g^ LGL, low-grade squamous intraepithelial lesions and cervical intraepithelial lesions grade 1. ^h^ Invasive cervical cancer, squamous cell carcinoma, and high-grade lesions. ^i^ AC, adenocarcinoma and adenosquamous carcinoma. ^j^ More than one ethnic descent. ^k^ Exfoliated cervical cells and white blood cells.
