# Supplementary material for: Null Genotypes of GSTM1 and GSTT1 Contribute to Risk of Cervical Neoplasia: An Evidence-Based Meta-Analysis
Source: PLoS One. 2011 May 23;6(5):e20157. doi: 10.1371/journal.pone.0020157 (PMC3100325; doi:10.1371/journal.pone.0020157)
Supplement: Table S5 — Summary odds ratios with confidence intervals between the GSTT1 polymorphism and cervical neoplasia risk. (DOC) [file pone.0020157.s009.doc]

| *GSTT1* | n ^a^ | Cases/  controls | Heterogeneity | |  | Model for  meta-analysis ^c^ |  | Null versus present | |  | *P* _Egger’s test_ ^d^ |
| --- | --- | --- | --- | --- | --- | --- | --- | --- | --- | --- | --- |
|  |  |  | *I*^2^ (%) | *P* _heterogeneity_ ^b^ |  |  |  | OR (95%CI) | *P* |  |  |
| Total | 19 | 2,092/2,054 | 59.1 | 0.001 |  | R |  | 1.30 (1.05-1.62) | 0.017 |  | 0.616 |
| Pathologic types |  |  |  |  |  |  |  |  |  |  |  |
| Cervical cancer (unclear type) | 7 | 776/1,080 | 69.9 | 0.003 |  | R |  | 1.49 (1.02-2.19) | 0.040 |  | 0.826 |
| SCC ^e^ | 4 | 394/461 | 51.3 | 0.104 |  | R |  | 1.00 (0.60-1.65) | 0.984 |  | 0.605 |
| HGL ^f^ | 5 | 287/518 | 28.1 | 0.234 |  | F |  | 1.18 (0.85-1.66) | 0.326 |  | 0.275 |
| LGL ^g^ | 3 | 179/264 | 0.0 | 0.402 |  | F |  | 1.05 (0.68-1.61) | 0.824 |  | 0.253 |
| Mixed ^h^ | 2 | 271/290 | 56.1 | 0.131 |  | R |  | 1.26 (0.67-2.37) | 0.470 |  | - |
| AC ^i^ | 2 | 54/189 | 78.1 | 0.033 |  | R |  | 1.97 (0.30-13.11) | 0.483 |  | - |
| SIL (unknown grade) ^j^ | 1 | 131/180 | - | - |  | R/F |  | 1.12 (0.69-1.81) | 0.644 |  | - |
| Ethnicities |  |  |  |  |  |  |  |  |  |  |  |
| Asian | 12 | 1,501/1,606 | 63.3 | 0.002 |  | R |  | 1.30 (1.00-1.71) | 0.053 |  | 0.800 |
| Caucasian | 5 | 417/632 | 0.0 | 0.614 |  | F |  | 1.05 (0.78-1.43) | 0.737 |  | 0.640 |
| Mixed ^k^ | 1 | 131/180 | - | - |  | R/F |  | 1.12 (0.69-1.81) | 0.644 |  | - |
| Unknown | 1 | 43/86 | - | - |  | R/F |  | 4.58 (2.04-10.28) | <0.001 |  | - |
| Source of DNA for genotyping |  |  |  |  |  |  |  |  |  |  |  |
| White blood cells | 12 | 1,432/1,659 | 63.2 | 0.002 |  | R |  | 1.24 (0.95-1.61) | 0.108 |  | 0.636 |
| Exfoliated cervical cells | 3 | 252/327 | 0.0 | 0.768 |  | F |  | 1.36 (0.89-2.08) | 0.161 |  | 0.793 |
| Mixed ^l^ | 2 | 190/251 | 66.3 | 0.085 |  | R |  | 2.81 (1.14-6.94) | 0.025 |  | - |
| No available data | 2 | 218/267 | 0.0 | 0.687 |  | F |  | 0.89 (0.58-1.37) | 0.582 |  | - |
| Quality criteria |  |  |  |  |  |  |  |  |  |  |  |
| Quality score ≥ 7 | 7 | 864/882 | 67.2 | 0.006 |  | R |  | 1.30 (0.90-1.87) | 0.162 |  | 0.315 |
| Quality score < 7 | 12 | 1,228/1,622 | 56.7 | 0.008 |  | R |  | 1.30 (0.99-1.72) | 0.064 |  | 0.273 |
| Matched (age) | 8 | 942/996 | 46.3 | 0.071 |  | R |  | 1.26 (0.96-1.67) | 0.097 |  | 0.265 |
| Unmatched (age) | 11 | 1,150/1,508 | 67.8 | 0.001 |  | R |  | 1.35 (0.97-1.89) | 0.076 |  | 0.387 |
| Smoking status |  |  |  |  |  |  |  |  |  |  |  |
| Smoking | 4 | 232/152 | 0.0 | 0.891 |  | F |  | 1.50 (0.96-2.36) | 0.076 |  | 0.291 |
| Non-smoking | 4 | 171/221 | 55.3 | 0.082 |  | R |  | 0.68 (0.33-1.39) | 0.290 |  | 0.198 |
| HPV infection status |  |  |  |  |  |  |  |  |  |  |  |
| HPV positive | 5 | 320/139 | 69.3 | 0.011 |  | R |  | 1.49 (0.59-3.78) | 0.400 |  | 0.278 |
| HPV negative | 4 | 178/288 | 0.0 | 0.741 |  | F |  | 0.80 (0.49-1.30) | 0.364 |  | 0.775 |

^a^ Number of studies. ^b^ *P* _heterogeneity_, *P* value of Q-test for heterogeneity test. ^c^ R, random-effects model; F, fixed-effects model. ^d^ *P* _Egger’s test_, the *P* value for Egger’s test. ^e^ SCC, squamous cell carcinoma. ^f^ HGL, high-grade squamous intraepithelial lesions and cervical intraepithelial lesions grades 2 and 3. ^g^ LGL, low-grade squamous intraepithelial lesions and cervical intraepithelial lesions grade 1. ^h^ Cervical cancer and high-grade lesions. ^i^ AC, adenocarcinoma and adenosquamous carcinoma. ^j^ Squamous intraepithelial lesions. ^k^ More than one ethnic descent. ^l^ Exfoliated cervical cells and white blood cells.
