# Supplementary material for: Null Genotypes of GSTM1 and GSTT1 Contribute to Risk of Cervical Neoplasia: An Evidence-Based Meta-Analysis
Source: PLoS One. 2011 May 23;6(5):e20157. doi: 10.1371/journal.pone.0020157 (PMC3100325; doi:10.1371/journal.pone.0020157)
Supplement: Table S6 — Summary odds ratios with confidence intervals between the GSTM1-GSTT1 interaction and cervical neoplasia risk. (DOC) [file pone.0020157.s010.doc]

| *GSTM1-GSTT1* interaction | n ^a^ | Cases/  controls | Heterogeneity | |  | Model for  meta-analysis ^c^ |  | Null versus present | |  | *P* _Egger’s test_ ^d^ |
| --- | --- | --- | --- | --- | --- | --- | --- | --- | --- | --- | --- |
|  |  |  | *I*^2^ (%) | *P* _heterogeneity_ ^b^ |  |  |  | OR (95%CI) | *P* |  |  |
| Null/null versus present/present | 4 | 499/503 | 20.5 | 0.287 |  | F |  | 1.72 (1.18-2.51) | 0.004 |  | 0.176 |
| Null/null versus present/null | 4 | 499/503 | 0.0 | 0.489 |  | F |  | 1.32 (0.92-1.90) | 0.130 |  | 0.271 |
| Null/null versus null/present | 4 | 499/503 | 0.0 | 0.420 |  | F |  | 1.45 (1.00-2.11) | 0.051 |  | 0.349 |
| Null/present versus present/null | 4 | 499/503 | 61.9 | 0.049 |  | R |  | 1.06 (0.57-1.97) | 0.855 |  | 0.060 |
| Null/present versus present/present | 4 | 499/503 | 34.9 | 0.203 |  | F |  | 1.20 (0.84-1.70) | 0.311 |  | 0.726 |
| Present/null versus present/present | 4 | 499/503 | 68.6 | 0.023 |  | R |  | 1.07 (0.54-2.11) | 0.841 |  | 0.074 |

^a^ Number of studies. ^b^ *P* _heterogeneity_, *P* value of Q-test for heterogeneity test. ^c^ R, random-effects model; F, fixed-effects model. ^d^ *P* _Egger’s test_, the *P* value for Egger’s test.
